# Deep transcriptome analysis using RNA-Seq suggests novel insights into molecular aspects of fat-tail metabolism in sheep

**DOI:** 10.1038/s41598-019-45665-3

**Published:** 2019-06-24

**Authors:** Mohammad Reza Bakhtiarizadeh, Abdolreza Salehi, Ali A. Alamouti, Rostam Abdollahi-Arpanahi, Seyed Alireza Salami

**Affiliations:** 1 Department of Animal and Poultry Science, College of Aburaihan, University of Tehran, Tehran, Iran; 2 University of Tehran, Tehran, Iran

**Keywords:** Gene expression profiling, Comparative genomics, Gene regulatory networks

## Abstract

Fat-tail content of sheep breeds is varied and the molecular mechanisms regulating fat-tail development have not been well characterized. Aiming at better identifying the important candidate genes and their functional pathways contributing to fat deposition in the tail, a comparative transcriptome analysis was performed between fat- (Lori-Bakhtiari) and thin-tailed (Zel) Iranian sheep breeds using RNA-seq. The experiment was conducted on six male lambs (three lambs per each breed) at seven months of age. Four different combinations of aligners and statistical methods including Hisat2 + edgeR, Hisat2 + DESeq2, STAR + edgeR and STAR + DESeq2 were used to identify the differentially expressed genes (DEGs). The DEGs were selected for functional enrichment analysis and protein-protein interaction (PPI) network construction. Module analysis was also conducted to mine the functional sub-networks from the PPI network. In total, 264 genes including 80 up- and 184 down-regulated genes were identified as DEGs. The RNA-Seq results were validated by Q-RT-PCR. Functional analysis of DEGs and the module analysis of PPI network demonstrated that in addition to pathways affecting lipid metabolism, a series of enriched functional terms related to “response to interleukin”, “MAPK signaling pathways”, “Wnt signaling pathway”, “ECM-receptor interaction”, “regulation of actin cytoskeleton”, and “response to cAMP” might contribute to the deposition of fat in tails of sheep. Overall results using RNA-Seq analysis characterized important candidate genes involved in the fatty acid metabolism and regulation of fat deposition, suggesting novel insights into molecular aspects of fat-tail metabolism in sheep. Selected DEGs should be further investigated as potential markers associated with the fat-tail development in sheep breeds.

## Introduction

Sheep are one of the most important livestock animals and are also known as the first domesticated grazing animal. Over 11,000 years of domestication and artificial selection have extremely diversified sheep breeds phenotypic characteristics^1^. It is believed that fat-tailed sheep were derived from thin-tailed sheep during the process of domestication, approximately 5,000 years ago^2^. In fat-tailed sheep, which account for one-fourth of the world’s sheep population^3^, the intensity of fat deposition (up to 20% of carcass weight) is higher in the tail than in other parts of the body^4^. In evolutionary terms, fat-tail was developed as a survival mechanism in hazardous environments and formed a reliable energy reservoir for the animal during drought and food deprivation periods^5^. Hence, fat-tail became a common characteristic in all sheep breeds distributed in semi-arid and arid regions of the world^6^. However, in todays’ modernized production systems, sheep are preferably kept under intensive or semi-intensive feeding systems where large fat-tails are no longer of historical importance as an energy source^7,8^. Conversely, fat deposition in the tail requires a greater energetic cost than accretion of an equivalent amount of lean tissue. Furthermore, the price of each kilogram of fat-tail hardly reaches 15% the price of each kilogram red meat thereby favoring production of breeds with a down-sized fat-tail^9^. Thus, the identification of the candidate genes and molecular pathways regulating fat deposition in the tail of sheep are economically important.

Long-term artificial selection along with geographical distribution has resulted in generation of over 28 indigenous sheep breeds with large fat-tails in Iran, except Zel breed which has a thin tail^10^. Lori–Bakhtiari is one of the relatively large frame breeds in the south-western parts of Iran possessing the largest fat-tail among all domestic sheep breeds^11^. In contrast, Zel is the only thin-tailed Iranian sheep breed (with 10–12 cm tail length) with a small body frame mainly distributed in the coastal area around the Caspian Sea^12^. Significant differences in fat deposition between these two breeds may allow for the identification of pathways potentially responsible for formation of tail fat, thereby assisting in the development of new breeding strategies to manipulate fat deposition. Previous studies have shown that tail fat metabolism in different sheep breeds is associated with mRNAs^13–17^, micro RNAs (miRNAs)^18,19^ and long non-coding RNAs^20,21^. For example, Wang *et al*.^13^, reported 646 differentially expressed genes (DEGs) by comparing transcriptome profiles of fat between a fat-tailed (Kazak sheep) and a short-tailed sheep (TS). They also suggested two important genes (NELL1 and FMO3) as important candidates in fat metabolism of sheep. In another study^14^, the transcriptome information of subcutaneous adipose tissue between Small Tailed Han and Dorset sheep was analyzed and 602 DEGs were reported, some of which were suggested to be involved in the fat metabolism process through functional analysis. Furthermore, recent studies on sheep have suggested important roles of developmental genes (such as HOXC11, HOXC12 and HOXC13), fat-tailed expressed genes (such as HOTAIR_2, HOTAIR_3 and SP9)^16^ and fat-related genes (FABP4, FABP5, ADIPOQ, and CD36)^17^ in fat deposition. However, few studies have been conducted to identify important functional genes or selective genomic regions associated with tail fat formation by comparing differences between these two extremely different Iranian sheep breeds. Moradi *et al*.^7^ investigated selection signatures in these sheep breeds and identified three regions located on chromosomes 5, 7 and X linked to the formation of tail fat. Also, in a previous study, we applied a comparative genomic approach and introduced fatty acid binding protein 4 (FABP4) as a candidate gene relevant to fat deposition in the tail of Lori–Bakhtiari^8^. Mohammadi *et al*.^22^ showed that a silent mutation in exon 17 of the diacylglycerol acyltransferase 1 (DGAT1) gene had a positive effect on tail fat weight and backfat thickness in Lori-Bakhtiari compared to Zel breed. Nevertheless, the genetic factors underlying the formation of tail fat on a genome-wide level remain to be elucidated in Iranian sheep breeds.

Gene expression profiling by RNA sequencing (RNA-Seq) provides an opportunity to better understand the underlying mechanisms of fat deposition. To date, several studies have applied this approach to compare transcriptomes in various fat depots in humans^23^, pigs^24^ and cattle^25^. Moreover, there have also been a few comparative transcriptome studies between different thin and fat-tailed sheep breeds^14,24^. However, to the best of our knowledge, there has been no genome-wide gene expression profiling study specifically comparing the transcriptome between fat- and thin-tailed sheep breeds. Hence, in this study we compared Lori-Bakhtiari and Zel breeds at the transcriptome level using RNA-Seq technology to identify the determinant genes as well as the potential underlying molecular mechanisms governing the fat deposition in sheep tails.

## Materials and Methods

### Ethics statement and experimental design

All animal care and experiments were approved by the research council of the University of Tehran, Iran. Also, all experiments were performed in accordance with a routine guideline which is acceptable by the research council of the University of Tehran. Three male lambs (weaned at the age of 90 days on average) from each purebred Lori-Bakhtiari and Zel were used in this study. All animals were reared under the same environmental conditions at the research station of the college of Aburaihan, University of Tehran. Also, lambs were individually fed on the same nutritional conditions with ad libitum access to food and water. Adipose tissue samples from the tail fat of the sheep were collected immediately after the animals were slaughtered at seven months of age. All tissue samples were snap frozen in liquid nitrogen and then transferred to a −80 °C freezer until required for RNA isolation.

### RNA Extraction and transcriptome sequencing

Total RNA was isolated from approximately 100 mg of adipose tissue samples using the Tripure isolation reagent kits (Roche Applied Science), following the manufacturer’s instructions. RNA was quantified using the NanoDrop (Thermo Scientific™ NanoDrop 2000) and purity of samples was checked on 1% agarose gels for evaluating the 28S and 18S ribosomal RNA bands (28S/18S ratio). All samples with a ratio (28S/18S) of above 1.8 and an OD 260/280 ratio greater than 1.9 were sent to BGI Company in China for sequencing. RNA integrity number (RIN) was also measured on an Agilent Bio Analyzer 2100 system. Only RNA samples with a RIN > 7 were used for cDNA library construction. All cDNA libraries were sequenced using paired-end strategy (read length 150 bp) on an Illumina HiSeq 2000 platform. The raw RNA-Seq data were deposited and released in SRA database, with the BioProject accession number of PRJNA508203.

### Quality control and read trimming

A quality check of the raw sequencing data was performed using FastQC (v0.11.5) program to detect common issues in RNA-Seq data. The reads were then trimmed with Trimmomatic to remove low quality bases using the options: TRAILING:20, MAXINFO:120:0.9, MINLEN:120. The quality of the reads was re-assessed with FastQC after this step to confirm quality improvements.

### Mapping to genome and identification of DEGs

Alignment of cleaned reads to the genome is the first step in the majority of RNA-Seq studies in model organisms with a reference genome. The next very important step is normalization and statistical modeling to identify DEGs. In recent years, many alignment tools and normalization/statistical methods have been developed. Also, several benchmark studies have been conducted to assess the performance of these methods. Results of these studies showed that there is no single method/tool in each step that outperforms all other methods on all possible conditions^26^. It has been demonstrated that choice of alignment tool is critical for accurate interpretation of RNA-Seq data^26,27^ as well as the method of normalization or statistical analysis has the strongest impact on performance^28^. Hence, benchmark studies showed that a combination of different methods in each step can be an efficient way to achieve more reliable results^28^. In the present study, to improve the accuracy of the identified DEGs, we combined the results of two alignment tools (Hisat2 and STAR) as well as two statistical methods (edgeR and DESeq2). Therefore, we investigated four different combinations (1) Hisat2 + edgeR, (2) Hisat2 + DESeq2, (3) STAR + edgeR and (4) STAR + DESeq2. These tools were chosen based on the literature confirmation of their robustness and high efficiency^26,29^. Finally, only those genes that were identified as differentially expressed by the four approaches, were considered as DEGs and were subjected to further analysis. This combined approach was used to ensure the exclusion of false positives.

Clean reads were aligned to reference sheep genome (Oar_v3.1) using STAR (version 2.5.3a)^30^ with the following parameters:–outFilterType BySJout, –outFilterMismatchNmax 10, –sjdbOverhang 100, –outFilterMultimapNmax 10 and–outFilterScoreMin 0. Additionally, Hisat2 software (version 2.1.0)^31^ was run with default parameters. The sequence alignment files generated by STAR and Hisat2 were used as the input to HTSeq-count (Python package HTSeq, python v 2.7.3) software^32^ to generate counts of uniquely mapped reads to annotated genes using the reference annotation (version 88) file. The reference genome and the reference annotation for the sheep were obtained from the Ensembl database (http://asia.ensembl.org/info/data/ftp/index.html).

The genes that differed significantly between two breeds were identified with two count based methods in R packages including edgeR (version 3.18.1)^33^ and DESeq2 (version 1.16.1)^34^. In other words, the obtained raw gene-count table for each of the alignment tools was used as input in these packages, separately. Both packages are currently considered robust methods for differential expression analysis using a generalized-linear model and are based on the negative binomial distribution. However, they mainly differ in their approaches to estimate the dispersion parameter and to normalize raw counts. DESeq2 obtains dispersion estimates based on calculated mean–variance relationships in the given data set, while edgeR assumes a common dispersion for all the genes. Here, in the edgeR analysis, normalization was performed using the trimmed mean of M values (TMM) method and the dispersion parameter for each gene was calculated as the Cox-Reid common dispersion method. In DESeq2 analysis, a normalization factor was estimated using the median-of-ratios method. Dispersions were then estimated using a Cox-Reid adjusted profile likelihood. In both methods Benjamini-Hochberg correction was used to correct for multiple comparisons (with a false discovery cut-off <0.05). As mentioned above, the cross-validated results of all the four pipelines were considered as a final set of the DEGs. Furthermore, a principal component (PCA) analysis was performed using SARTools^35^ (using DESeq2 package) for clustering the samples based on gene expression patterns in order to examine the level of similarity/dissimilarity in the gene expression profiles of the two breeds.

### Functional analysis

Enrichr web-based tool^36^ was used to perform gene set enrichment analysis. This analysis was performed to provide more information about the biological functions and pathways significantly enriched in up- or down-regulated genes (DEGs confirmed by the four pipelines) by focusing on gene ontology (GO) terms (BP, biological process) and Kyoto Encyclopedia of Genes and Genomes (KEGG) pathways (using a standard false discovery rate (FDR) < 0.05).

### Construction of PPI network and screening of sub-networks

Considering the protein-protein interactions (PPI) in the complex biological systems, STRING (the Search Tool for the Retrieval of Interacting Genes/Proteins) database (version 10.5) was explored to reveal functional interactions among the DEGs. The STRING database collects and integrates all of functional interactions between the genes/proteins by consolidating known and predicted protein–protein association data for a large number of organisms^37^. PPIs with confidence score <0.4 (a commonly used threshold) was discarded and disconnected nodes were hidden. ClusterONE plugin (Clustering with Overlapping Neighborhood Expansion, version 1.0) in Cytoscape was applied to detect the sub-network/modules in the PPI network, provided by STRING database. A cut-off value of P ≤ 0.01 and a minimum number of genes in a cluster >5 were utilized to measure the significance of the predicted modules. ClusterONE uses a greedy growth process to detect densely connected sub-networks/modules of a PPI network^38^. The functional enrichment analysis for the identified significant modules was further performed using Enrichr database. These modules were considered as candidate functional modules if their respective genes were significantly enriched in GO or KEGG pathways. Cytoscape software (version 3.6) was applied to visualize the PPI networks^39^.

### Q-RT-PCR validation of data

In order to validate the RNA-Seq data, 10 genes were randomly picked from DEGs pool and were tested with Q-RT-PCR using a new set of RNAs extracted from the same fat-tail tissues as that for RNA-Seq analysis along with four new lambs (including two Lori-Bakhtiari and two Zel breeds). Four new sheep breeds were reared under the same environmental conditions along with the six sheep which were applied in RNA-Seq assay. First-strand cDNA,s were synthesized using the first strand cDNA synthesis kit (Thermo Fisher, Co., USA) according to the manufacturer’s instructions. Primers were designed using Primer3Plus software^40,41^. The primer sequences are listed in Supplementary File 1. All Q-RT-PCR reactions were run on Light-Cycler 96 instrument (Roche Co. Germany) using the SYBR Green Master Mix (Thermo Fisher Scientific, USA), according to the manufacturer’s protocol. Q-RT-PCR was performed with five biological replicates and three technical replicates for each sample. C_T_-method was used to quantify changes in gene expression, while glyceraldehyde-3-phosphate dehydrogenase (*GAPDH*) was used as housekeeping gene reference standard. The selection of the endogenous control was mainly based on our previous study^8^. The relative expression of each gene was determined based on comparative delta-delta C_T_ method (2^−ΔΔCT^). To compare Q-RT-PCR data with RNA-Seq data, the mean of 2^−ΔΔCT^ value of each gene was converted into a fold change.

## Results

### Descriptive statistics of RNA-Seq data

**Figure 1:**
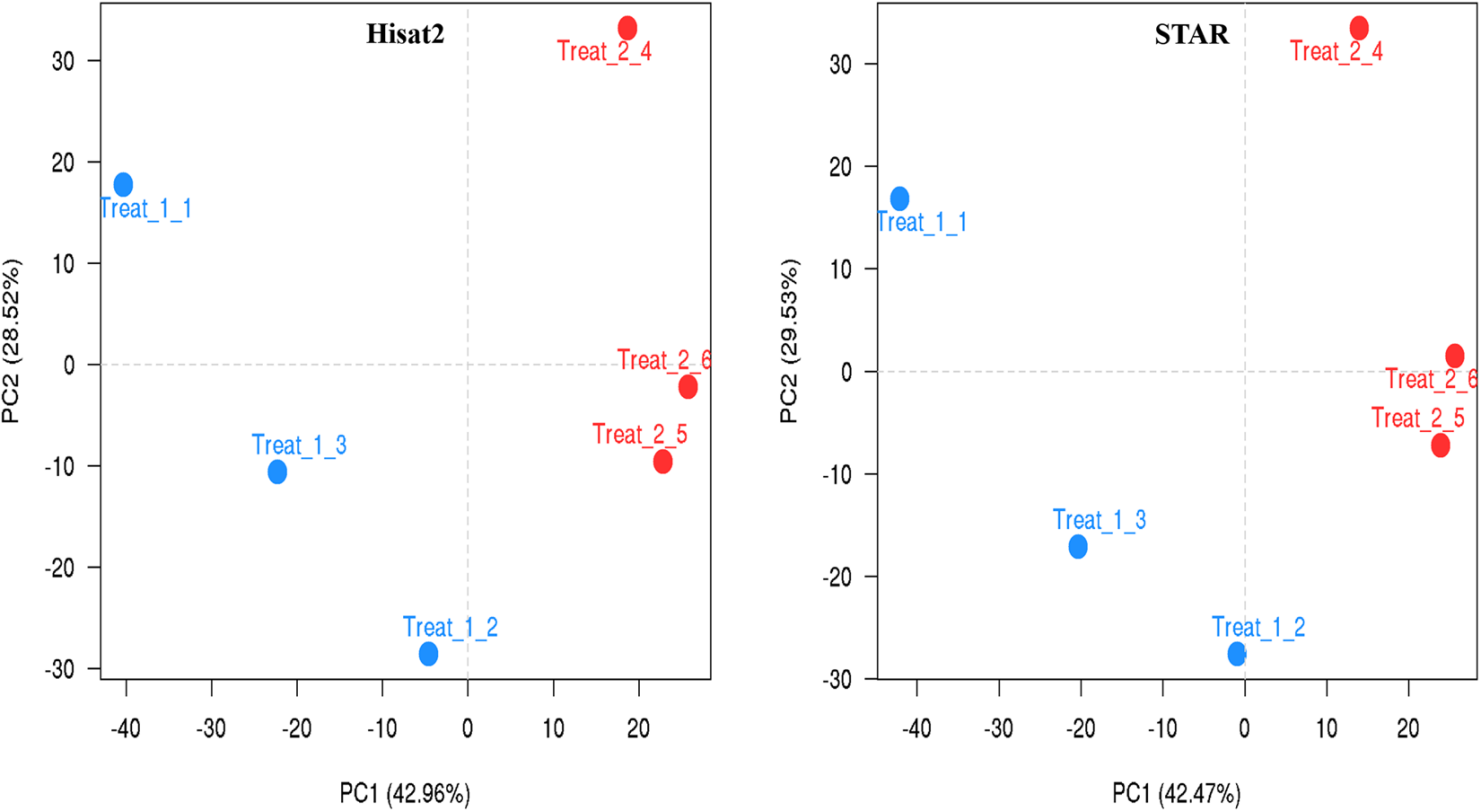
PCA scatter plot of gene expression in the sheep breeds. Left and right plots were obtained based on Hisat2 and STAR tools results, respectively. Treat_1 and Treat_2 indicate Lori-Bakhtiari and Zel breeds, respectively. PCA plot showing variance of the three biological replicates of each of the two sheep breeds. The percentages on each axis represent the percentages of variation explained by the principal components.

In total, 129,776,763 paired-end raw reads (150-bp in length) were generated for six samples, ranging from 20.1 to 26.3 million per sample. Among these, 66.8 and 62.9 million reads were belonged to Lori-Bakhtiari and Zel breeds, respectively. On average, individual samples yielded 21.6 million (±2.4) reads. Only 1,104 reads were removed after quality trimming and filtration, indicating that data had high quality. To align the clean reads to reference genome, we employed two state-of-the-art and widely used aligners, Hisat2 and STAR. On average, 86 and 85% of all the clean reads were aligned to reference genome by Hisat2 and STAR tools, respectively. Moreover, 76% of all the clean reads were uniquely mapped to the reference genome by each of the aligners. The summary of the RNA sequencing and mapping of the six samples are presented in Table 1. PCA analysis was performed with the normalized counts (based on the DESeq2 method) to investigate if samples from the same breed cluster together. As a result, the first two principal components (PCs) explained more than 70% of the variability among the samples and both of the breeds were grouped in distinct clusters (Fig. 1). This finding indicated a clear difference between the transcriptome profiles of two breeds. The Lori-Bakhtiari samples fell in the negative, whereas the Zel samples fell in the positive direction of the PC1 axis. In PC2, one biological replicate of each breed did not cluster with the others likely due to individual variability between the animals. Therefore, the differences in gene expression profiles would enable us to identify candidate genes explaining the known differences in the fat-tail shape that exist between these two breeds.

**Table 1:** Descriptive statistics of sequence quality and mapping rate from Lori-Bakhtiari and Zel sheep breeds.

Breeds	Raw reads	Trimmed reads	Hisat2 total mapping (%)	STAR total mapping (%)	Hisat2 uniquely mapped reads (%)	STAR uniquely mapped reads (%)
Lori-Bakhtiari_1	26,282,890	26,282,599	24,072,724 (92)	24,450,510 (93)	22,408,851 (85)	22,939,577 (87)
Lori-Bakhtiari_2	20,075,866	20,075,798	16,649,703 (83)	16,440,090 (82)	15,633,869 (78)	15,446,519 (77)
Lori-Bakhtiari_3	20,428,424	20,428,247	16,879,641 (83)	16,505,913 (81)	15,676,435 (77)	15,393,098 (75)
Zel_1	22,292,871	22,292,640	19,681,007 (83)	19,906,545 (89)	17,456,499 (78)	17,789,855 (80)
Zel_2	20,164,542	20,164,351	16,822,308 (83)	16,448,756 (82)	15,650,133 (78)	15,378,114 (76)
Zel_3	20,532,170	20,532,024	17,843,736 (87)	17,431,817 (85)	16,127,823 (79)	15,786,537 (77)

### Differential expression analysis

**Figure 2:**
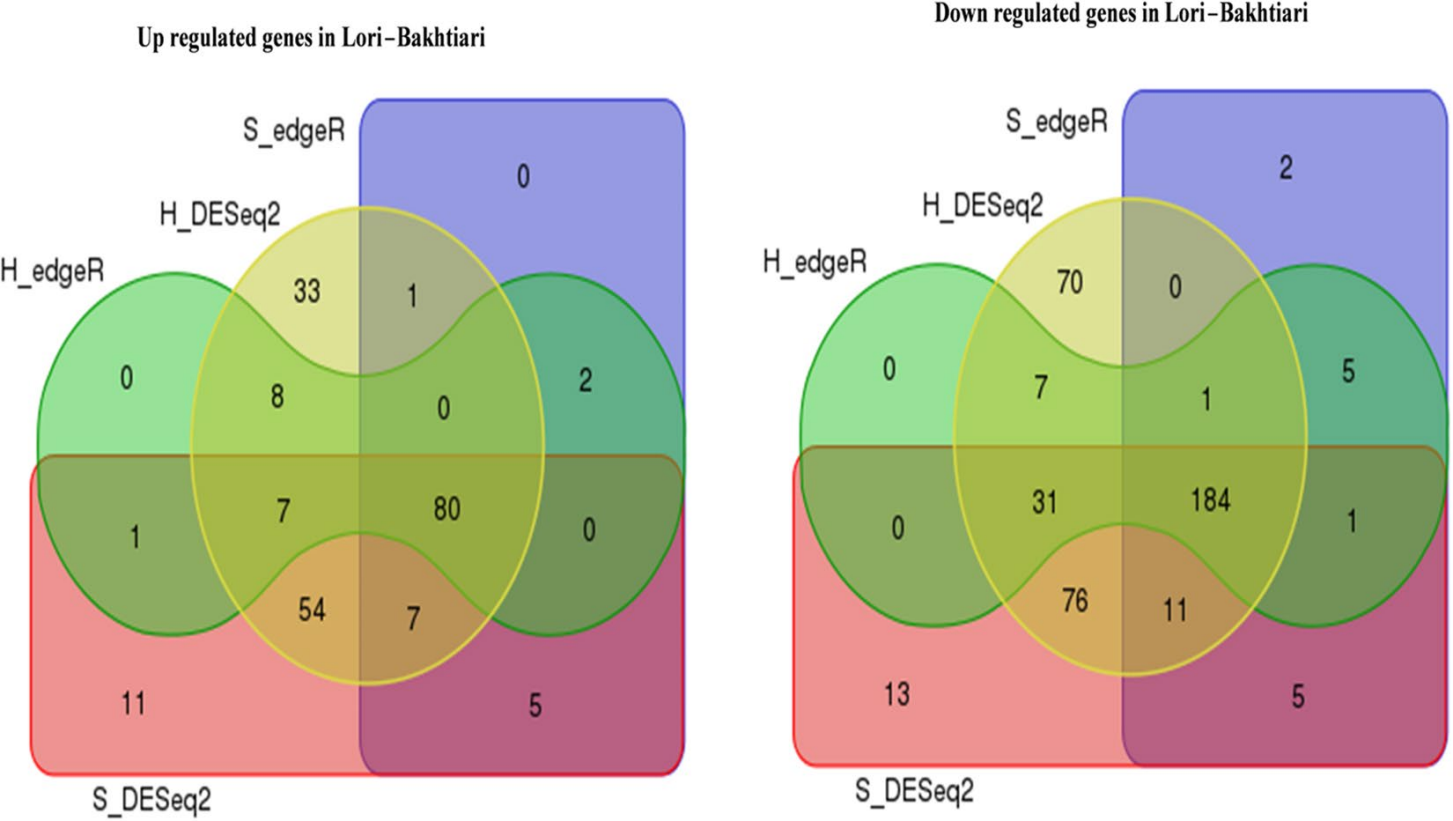
Venn diagram of the overlap in up-regulated and down-regulated DEGs among four methods. S and H represent the Hisat2 and STAR aligners, respectively.

**Figure 3:**
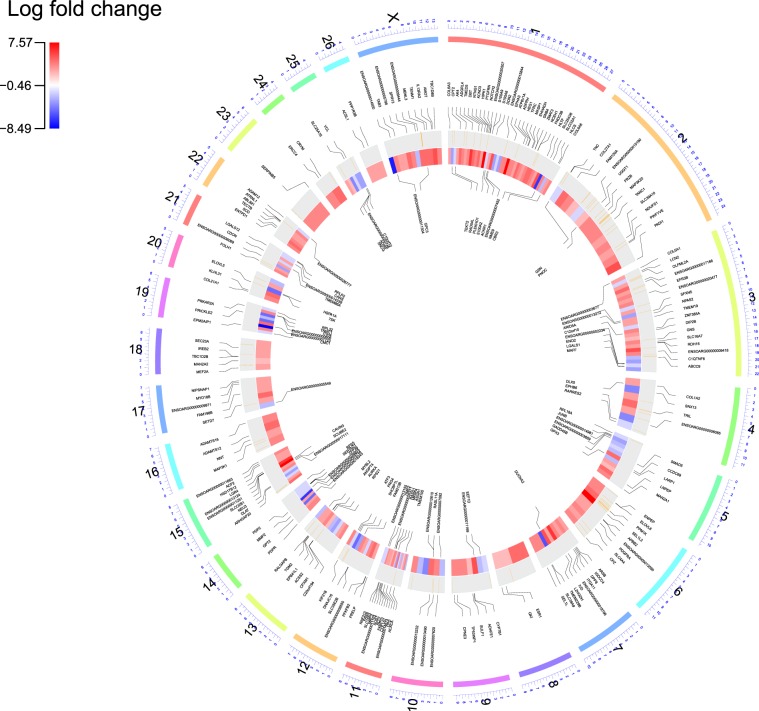
Circos plot of genomic distribution of DEGs in the sheep. The outer layer shows chromosome numbers. The positions of the DEGs are shown in the inner light gray layer. Also, the vertical orange lines in the inner light gray rings represent the position of the QTLs associated with lipid. The heatmap display the expression levels of each DEGs, as red and blue colors indicate down- and up-regulated genes in Lori-Bakhtiari breed, respectively.

We applied two well-established statistical analysis methods (edgeR and DESeq2) based on read counts to compare the expression levels in two under investigated breeds. Therefore, four approaches were used; Hisat2 + edgeR, Hisat2 + DESeq2, STAR + edgeR and STAR + DESeq2 identifying 327, 570, 304 and 486 DEGs, respectively. In total, 615 DEGs were found, out of which 209 were up-regulated while 406 were down-regulated in the Lori-Bakhtiari compared to the Zel. Amongst these DEGs, 607 were identified by DESeq2-based methods (~99%), indicating that DESeq2 is over-sensitive. The lowest number of singleton genes (defined as genes identified by only one method) was observed by the edgeR method in terms of both up- and down-regulated genes. Hisat2 + DESeq2 and STAR + DESeq2 methods returned 33 and 11 up-regulated as well as 70 and 13 down-regulated DEGs respectively, that the other methods did not find significant (adjusted-p < 0.05). Moreover, Hisat2 + DESeq2 and STAR + DESeq2 methods both predicted 54 and 76 up- and down-regulated genes not identified as DEGs by the two other methods, respectively (Fig. 2). Here, the overlap of DEGs among all four methods was considered for both up-regulated and down-regulated genes summing to 264 DEGs. Amongst these, 80 DEGs were up-regulated and 184 DEGs were down-regulated in in Lori-Bakhtiari breed (Fig. 2). A number of 50 and 173 up- and down-regulated DEGs (related to Lori-Bakhtiari breed) had log2 fold-change greater than 2 in expression levels, respectively, which accounted for 84% of the DEGs. The log2 fold-change for the commonly identified DEGs ranged from 1.5 to 8.5 (based on Hisat2 + edgeR method). Table 2 lists the top 10 known up- and down-regulated DEGs, based on their levels of significance. The details of differential gene expression analysis can be found in Supplementary File S2. The volcano plot of DEGs between two breeds for all four methods is provided in Supplementary File S3. Moreover, a Circos plot was generated to visualize the expression pattern and distribution of the DEGs on the sheep genome (Fig. 3).

**Table 2:** Top 10 known up-regulated and down-regulated DEGs in Lori-Bakhtiari compared to Zel breed based on Hisat2 + edgeR method.

Up-regulated DEGs			Down-regulated DEGs		
Gene name	FDR	Log2 fold change	Gene name	FDR	Log2 fold change
Troponin C type 1 (TNNC1)	3.33E-08	4.95	Stearoyl CoA Desaturase (SCD)	6.5E-08	3.81
NME/NM23 family member 9 (NME9)	9.7E-07	6.59	C1q and tumor necrosis factor related protein 6 (C1QTNF6)	4.09E-07	4.88
synuclein-γ (SNCG)	0.0001	4.02	Family with sequence similarity 198 member B (FAM198B)	3.54E-06	3.80
C10orf10	0.0002	3.57	Collagen type 1 α1 (COL1A1)	3.54E-06	3.83
Signal peptide-CUB-EGF domain-containing protein 2 (SCUBE2)	0.0003	3.33	SEL1L family member 3 (SEL1L3)	3.54E-06	7.09
Ras-like protein family member 11A (RASL11A)	0.0003	4.08	Peptidyl arginine deiminase 1 (PADI1)	3.54E-06	5.08
7SK	0.0006	4.58	Solute carrier family 25 member 36 (SLC25A36)	6.99E-06	4.53
Proteoglycan 4 (PRG4)	0.0011	5.18	Netrin G1 (NTNG1)	7.16E-06	5.34
Dual oxidase maturation factor 2 (DUOXA2)	0.0012	5.77	Crystallin mu (CRYM)	1.48E-05	4.075
Aurora kinase A (AURKA)	0.0012	3.24	EPH receptor A3 (EPHA3)	2.89E-05	7.016

### Functional enrichment analysis

**Figure 4:**
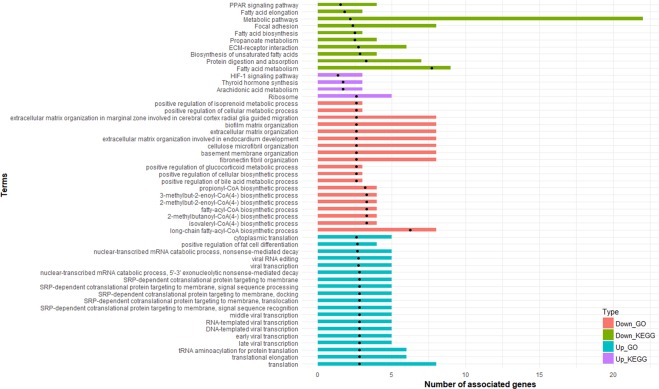
Results of functional enrichment analysis. Owing to the large number of significant GO terms, only the top 20 significant terms are displayed. Dot black points indicate -Log10 of FDR for each term. Up_GO: GO analysis results for up-regulated DEGs, Down_GO: GO analysis results for down-regulated DEGs, Up_KEGG: KEGG pathway analysis results for up-regulated DEGs and Down_KEGG: KEGG pathway analysis results for down-regulated DEGs.

To gain further insight into the metabolic processes differing between two breeds, functional enrichment analysis was performed (based on biological process GO terms and KEGG pathways) using up- and down-regulated DEGs. In total, 184 down-regulated DEGs were annotated in 83 significant GO terms, most of which were involved in biological processes related to lipid metabolism such as “long-chain fatty-acyl-CoA biosynthetic process”, “fatty acid biosynthetic process”, “lipid biosynthetic process”, “long-chain fatty acid biosynthetic process” and “glycerolipid biosynthetic process”. The KEGG pathway analysis of these genes identified 10 significant pathways (adjusted p < 0.05), which had similar patterns to GO terms, such as “fatty acid metabolism”, “biosynthesis of unsaturated fatty acids” and “fatty acid elongation”. On the other hand, GO analysis suggested that 80 up-regulated DEGs in Lori-Bakhtiari were significantly enriched in 93 processes, of which 15 terms were associated with “response to interleukin”. Most importantly, some of the significantly enriched GO terms were closely associated with lipid metabolism such as “positive regulation of fat cell differentiation” and “positive regulation of brown fat cell differentiation”, which might be of great interest in this dataset. Also, four KEGG pathways were significantly enriched for these genes including “ribosome”, “arachidonic acid metabolism”, “thyroid hormone synthesis” and “HIF-1 signaling pathway”. Figure 4 shows the complete list of enriched KEGG pathways and the top 20 significant GO terms in different datasets. The complete list of GO terms and KEGG pathways are listed in Supplementary File S4.

### PPI network and module analysis

**Figure 5:**
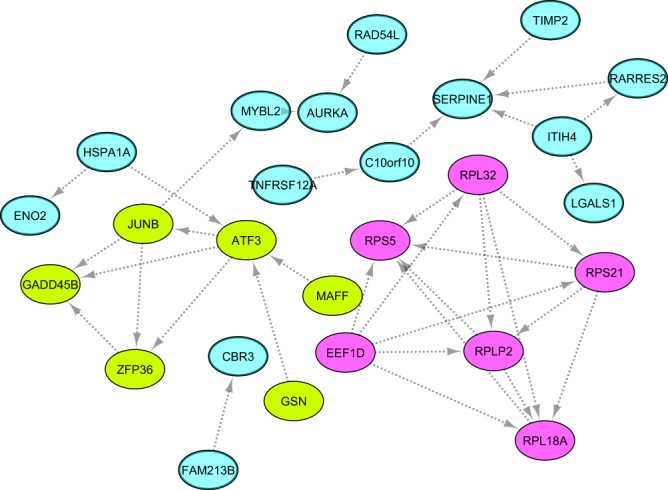
STRING-derived PPI network and the functional modules of up-regulated DEGs in Lori-Bakhtiari sheep breed. Pink nodes represent pink module (P-value = 0.0006) and light-green nodes denote the light-green module (P-value = 0.004).

**Figure 6:**
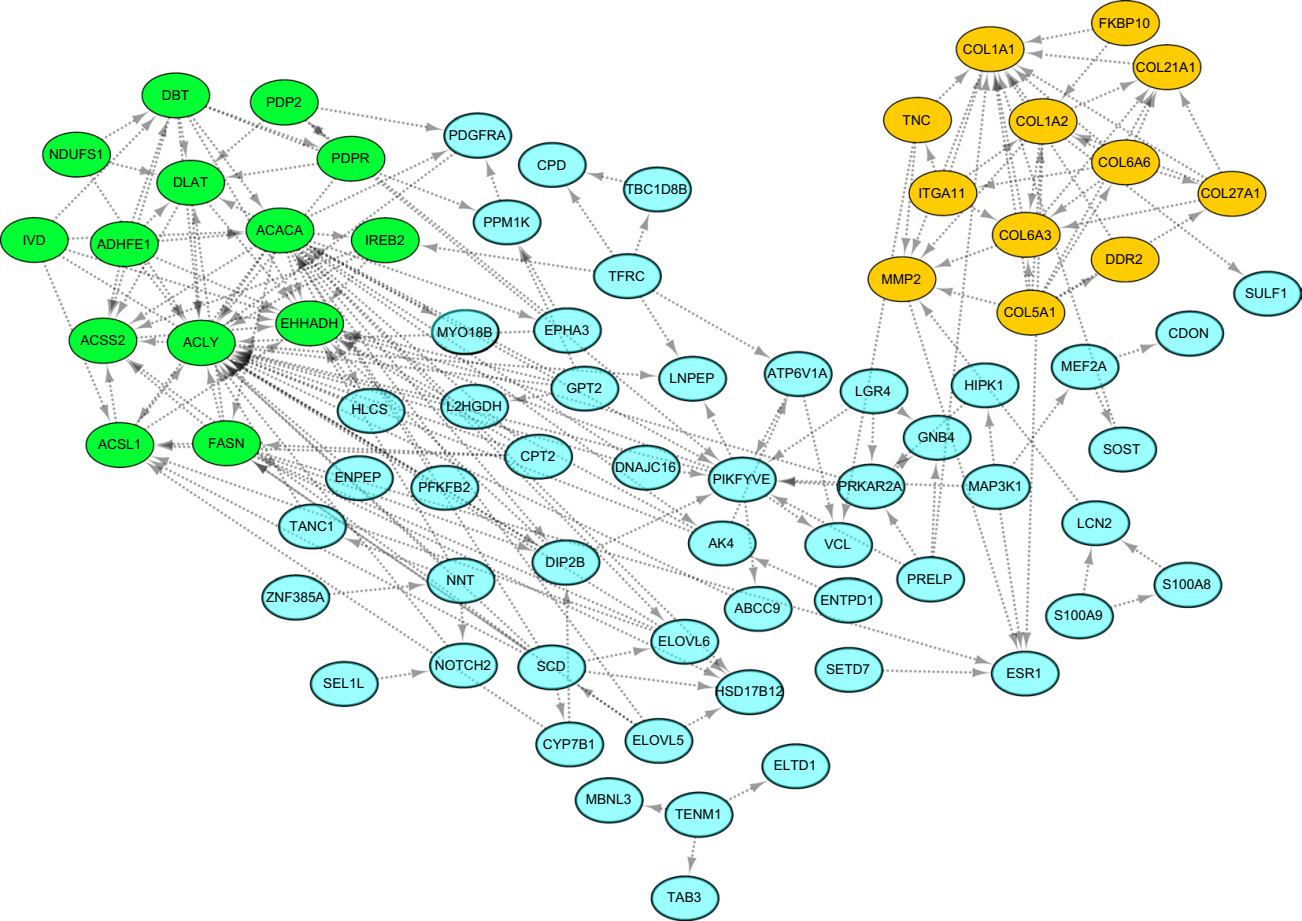
STRING-derived PPI network and the functional modules of down-regulated DEGs in Lori-Bakhtiari sheep breed. Dark-green nodes indicate dark-green module (P-value = 0.01) and orange nodes represent the orange module (P-value = 0.00002).

To establish DEGs form interactive PPI networks as well as to find out the functional relationship among up- and down-regulated DEGs, these genes were imported to STRING tool, separately. STRING can identify a network of close interactions among this set of genes based on databases of experimental and predicted protein interactions^37^. Of 264 DEGs, 218 genes were annotated, including 57 and 161 up- and down-regulated DEGs. Of these, 57 and 159 proteins associated with these genes, were matched with the database and used to construct the PPI network (Figs 5 and 6). A total of 208 known or predicted interactions (edges, PPI enrichment p-value = 2.69e-05) were formed among 26 up-regulated DEGs in Lori-Bakhtiari (Fig. 5). Also, a total of 92 genes formed a tightly connected network with 208 edges (PPI enrichment p-value = 0) in down-regulated DEGs (Fig. 6). The statistical enrichment analysis incorporated in STRING showed that the PPI networks were significantly enriched (adjusted-p < 0.05). It has been accepted that clustering algorithms is useful for grouping of proteins into functional modules^42^. Therefore, functional modules were extracted from PPI networks using ClusterONE algorithm. Two functional modules were identified with six nodes in up-regulated DEGs in Lori-Bakhtiari breed, designated as pink module (P-value = 0.0006, Fig. 5) and light-green module (P-value = 0.004, Fig. 5). Functional enrichment analysis indicated that genes in pink module were significantly enriched in 54 GO terms and one KEGG pathway, which were related to translation, tRNA and rRNA processing. Also, 289 GO terms and one KEGG pathway were enriched in genes of light-green module, which were mainly related to lipid metabolism pathways such as “positive regulation of fat cell differentiation” and “regulation of lipid transport”. Furthermore, two significant modules including 12 (orange module in Fig. 6, P-value = 0.00002) and 14 (dark-green module in Fig. 6, P-value = 0.01) nodes were found in the down-regulated DEGs in Lori-Bakhtiari breed. These modules were also involved significant GO terms and KEGG pathways including 118 GO terms and 9 KEGG pathways in orange module, and 169 GO terms and 18 KEGG pathways in dark-green module. Approximately 59% of the genes in orange module were members of the collagen family. An orange module involved significant functional terms such as “extracellular matrix disassembly”, “protein digestion”, “absorption” and “focal adhesion”. On the other hand, nodes in dark-green module were mainly enriched in functional terms related to lipid metabolism such as “fatty acid biosynthetic process”, “lipid biosynthetic process”, “fatty acid biosynthesis” and “PPAR signaling pathway”. The detailed results of PPI network analysis, module identification and functional enrichment analysis for the modules are provided in Supplementary File S5.

### Validation of differentially expressed genes

**Figure 7:**
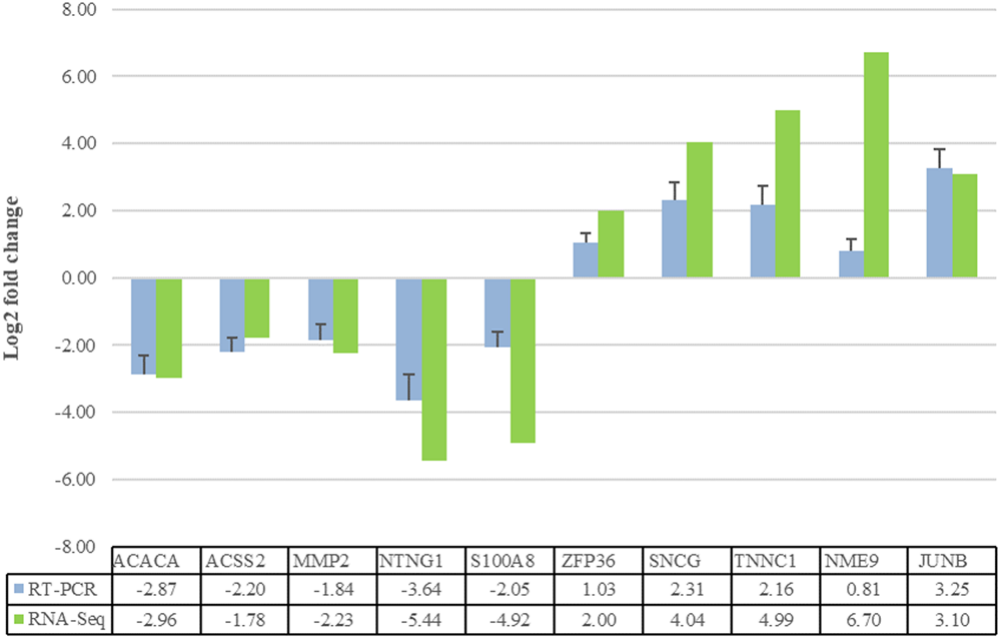
Q**-**RT-PCR validation of 10 randomly sampled genes identified by RNA-Seq analysis. Log2 fold expression change is relative to the mean expression level of the Lori-Bakhtiari breed.

To confirm the gene expression profile obtained by RNA-Seq analysis, 10 DEGs were examined by Q-RT-PCR. The ratio of the Log2 fold change from the RNA-seq analysis was compared to the Log2 fold change obtained with Q-RT-PCR. The mean expression values of Q-RT-PCR for each breed were calculated from the results of five independent biological replications. Figure 7 shows a comparison between the results from Q-RT-PCR and RNA-Seq analysis. For all 10 selected genes, the expression pattern determined by Q-RT-PCR analysis was similar to those detected using RNA-Seq, confirming the reliability of the RNA-Seq data. The complete results of Q-RT-PCR analysis are provided in Supplementary File S6.

## Discussion

Here, our primary goal was to determine the possible molecular mechanisms underlying fat accumulation in the tail of Iranian sheep breeds to further develop a theoretical basis for new breeding strategies. To have more reliable results and to minimize false positives, four pipelines were compared. Results showed that 43% of DEGs were common to all four pipelines. Identification of 264 DEGs supported that the genetic profile of fat-tail tissue differed between two breeds. Out of 80 up-regulated DEGs in Lori-Bakhtiari breed, 13 genes (Supplementary File S7) have also been reported in similar studies as important DEGs in fat deposition in sheep^13,15,18,43^ and cattle^44^. Likewise, of 184 down-regulated DEGs, 34 genes (Supplementary File S7) showed the same expression profile in similar studies conducted in sheep^13–15,18,20,43^ and cattle^44^.

Functional enrichment analysis suggested that most of the DEGs were directly or indirectly involved in lipid metabolism related pathways. This implies that molecular mechanisms underlying fat deposition in the tail of sheep are controlled by the interactions occurring in a complex network of genes. Among the enriched GO terms and KEGG pathways in the up-regulated DEGs, “positive regulation of fat cell differentiation” and “positive regulation of brown fat cell differentiation” were directly related to lipid metabolism. Some of the up-regulated DEGs grouped under terms related to lipid metabolism were Zinger finger protein (ZFP36), JUNB proto-oncogene (JUNB), retinoic acid receptor responder 2 (RARRES2) and adipogenesis regulatory factor (ADIRF). ZFP36 is actively involved in suppressing the expression of cytokines which is a lipolytic factor (e.g. IL-6), thus prevents adipose cell lipid degradation and favors its accumulation^45^. It also prevents obesity and improves lipid metabolism^46^. JUNB is a transcription factor (TF), the role of which has been documented in lipid metabolism and adipocyte differentiation^47^. RARRES2 (chemerin) is an adipokine discoverd to target adipose tissue and regulate adipogenesis^48^, insulin secretion and release of triglycerides and cholesterol in sheep^49^. ADIRF has many roles in fat cell development and fatty acid metabolism^50^, as it promotes adipocyte differentiation by inducing peroxisome proliferator activated receptor gamma (PPARG) expression^51^. In the present study, samples of adipose tissue were taken at the end of the experimental period when adipocyte differentiation was expected to complete, therefore the high expression level in the fat-tailed breed adipocytes pointed to contribution of these DEGs in adipocyte metabolism. Among them, ZFP36 and JUNB were also members of the light-green module in PPI network (light-green nodes in Fig. 5), suggesting that up-regulation of these genes might have resulted in excessive fat deposition in tail fat of Lori-Bakhtiari breed. This finding is further supported with the presence of ZFP36, JUNB, as well as activating transcription factor 3 (ATF3) in functional enrichment of light-green module for “regulation of lipid transport”. Earlier studies also proved that ATF3 represses the expression of adiponectin receptors in adipocyte cells^52^ and inhibits differentiation of preadipocyte 3T3-L1 cells^53^. In addition to the enrichment of directly related lipid metabolism processes, the orange module was also enriched for “sterol regulatory element-binding protein (SREBP) signaling pathway”, reinforcing the functional importance of the genes in that module. The SREBPs regulate pathways of cholesterol and fatty acid biosynthesis (Supplementary File S5)^54^. These findings suggest significant possible roles of these candidate genes in regulating fat deposition, which might have ultimately been a key point that contributed in morphological diversity between the sheep breeds.

As explained, GO analysis showed that DEGs related to “response to interleukin” were up-regulated in the Lori-Bakhtiari breed. In addition to the role of some inflammatory interleukins as signals of immunity challenges, recent studies suggested an important role of interleukins in other biological functions, especially in lipid metabolism^55^. Interestingly, response to interleukin-6, 17, 18 and 32 was significantly enriched in GO analysis in our study, which agreed with previous findings that IL-6^44,56^, IL-17^57^ and IL-32^3^, may regulate lipid metabolism through direct actions on adipose tissues. In this study, three DEGs were grouped under these terms including JUNB, inter-alpha-trypsin inhibitor heavy chain family member 4 (ITIH4) and tissue inhibitor of metalloproteinases 2 (TIMP2). ITIH4 and TIMP2 have been characterized as obesity-associated genes in human^58,59^. Besides, TIMP2 has a dynamic role in differentiation of adipocytes by inhibition of metalloproteinases^60^. In ruminant studies, a significantly higher expression of TIMP2 has been found in adipose tissue of a fat-tailed (Kazakhstan) in comparison to thin-tailed (Tibeta) sheep breed^15^. Hence, these genes may interact with interleukins to regulate adipocyte differentiation and fat deposition in fat-tailed sheep breeds.

The other significant GO terms were “response to cAMP” and “regulation of cAMP-mediated signaling”. The role of cAMP signaling pathway is well known in regulation of both adipogenesis and lipid partitioning in white adipose tissue as well as lipid metabolism in liver^61^. Several DEGs related to these processes such as JUNB, MAF BZIP Transcription Factor F (MAFF), AT-rich interaction domain 5A (ARID5A) and MYB proto-oncogene like 2 (MYBL2) were up-regulated in Lori-Bakhtiari breed. ARID5 subfamily includes ARID5A and ARID5B. In human^62^ and mice^63^, ARID5B knockdown was connected to lipid metabolism defects. ARID5B was not detected in our study; however, ARID5A was up-regulated in Lori-Bakhtiari suggesting that this gene might have a similar function in fatty acid metabolism, possibly through the cAMP signaling pathway. A previous study reported higher expression of MYBL2 in fat-tail of Kazakhstan (fat-tailed) compared with Tibetan (thin-tailed) sheep breed^13^. It appears that all the aforementioned genes contribute to a network controlling fat accumulation in tail of Lori-Bakhtiari breed.

In Lori-Bakhtiari, an increased expression was observed in genes related to MAPK signaling pathways including growth arrest and DNA damage inducible beta (GADD45B), retinoic acid receptor responder 2 (RARRES2) and ZFP36. The participation of MAPK pathway in adipogenesis either directly^64^ or indirectly, through inducing the expression of these genes, has been highlighted in previous studies^45,46,48,49^. In our study, GADD45B and ZFP36 were members of light-green module in PPI network (Fig. 5). Similarly, Huang *et al*.^44^, reported that the MAPK signaling pathway was functionally enriched for the up-regulated DEGs in Wagyu (a cattle breed with high intramuscular fat) compared to Holstein breed. Enrichment of this pathways has also been reported in miRNA-associated mRNA targets in fat-tailed Kazakhstan versus thin-tailed Tibetan sheep breed^15^. Thus, results of the current study provide further evidence that MAPK signaling pathway is an important component contributing to modulation of gene network of fat deposition in tail tissue.

Most of the significantly enriched GO terms and KEGG pathways of up-regulated DEGs in the Zel breed were directly related to lipid metabolism as other previous studied^15,18,20,43^ (Fig. 4, Supplementary File S4). For example, “biosynthesis of unsaturated fatty acids” and “fatty acid elongation” were enriched through GO enrichment of Lanzhou (fat-tailed) vs. Tibetan (thin-tailed) sheep breeds^20^. DEGs belonging to these processes were included SCD, fatty acid synthase (FASN), acetyl-CoA carboxylase alpha (ACACA), carnitine palmitoyl transferase 2 (CPT2), ELOVL fatty acid elongase 5 and 6 (ELOVL5 and ELOVL6), acyl-CoA synthetase long-chain family member 1 (ACSL1), enoyl-CoA hydratase and 3-hydroxyacyl CoA dehydrogenase (EHHADH), hydroxysteroid 17-beta dehydrogenase 12 (HSD17B12) and ATP citrate lyase (ACLY). Here, the gene expression pattern of FABP4, FASN, SCD and lipoprotein lipase (LPL) was consistent with our previous study^8^. Similarly, SCD, FASN, ACACA, ELOVL6, HSD17B12 and ACLY were reported as down-regulated DEGs in adipose tissue of Han (fat-tailed) in comparison with Dorset (thin-tailed) sheep breed. A negative regulatory relationship between a lincRNA and ACACA was proposed to regulate fat deposition in sheep breeds in our previous work^21^.

A possible explanation for down-regulation of a few lipid metabolism related genes in Lori-Bakhtiari breed is the potential functions of these genes in fatty acid oxidation. For instance, “PPAR signaling pathway” was significant in down-regulated genes in Lori-Bakhtiari as well as in the dark-green module (Fig. 6) and it is well known that TFs of the PPARG are crucially responsible for the clearance of cellular lipids via the regulation of many genes involved in fatty acid oxidation^65^. This corresponded with a previous study that showed “PPAR signaling pathway” was enriched in DEGs between fat-tail tissue of Guangling Large Tailed and Small Tailed Han sheep breeds^43^. In the present study, CPT2, EHHADH and ACSL1 were enriched in this pathway. CPT2 is essential for fatty acid oxidation^66^ and is ubiquitously expressed in tissues requiring fatty acids as energy-producing substrates^67^. Moreover, higher fat content in the tail than in visceral in Tan sheep breed is attributed to down-regulation of this gene^16^. EHHADH encodes a bifunctional β-oxidation enzyme involved in the peroxisomal fatty acid oxidation^68^. ACSL1 prepares long-chain fatty acids for oxidation through addition of co-enzyme A^69^. As the latter enzyme also directs fatty acids toward deposition in the form of triglycerides, the co-expression of ACSL1 with other aforementioned genes in the tail of Zel might be indicative of preferential partitioning of ACSL1 to oxidative, rather than deposition pathways. In addition, inhibition of ACLY is associated with fatty acid oxidation^70^. Thus, it is likely that the higher expression of these genes were responsible for stimulation of fatty acid oxidation leading to reduced fat deposition in the tail of Zel breed, as previously suggested, to explain smaller tail fat of the Tibetan vs. Kazakhstan sheep^13^.

On the other hand, down-regulated genes in Lori-Bakhtiari might have been related to other pathways than fat deposition such as fat composition. As result, the fatty acid composition of tail fat between two sheep breeds might have been different. In support of this, a significant breed effect has been reported on fatty acid composition of tail fat^71^. Also, lower SCD expression was reported in Guangling Large Tailed than Small Tailed Han^43^ as well as in Han than in Dorset (thin-tailed) sheep^18^. Based on this, lower content of saturated fatty acids can be anticipated in fat-tailed breeds, as SCD catalyzes an essential step in desaturation of saturated fatty acids^43^. Also, it is reported that HSD17B12^18^ and ELOVL6^72^ (both act as elongases) are important genes for controlling the overall balance of fatty acid composition. Likewise, lack of an obvious relationship between FASN expression and fat contents have been reported in sheep^73^, pig^74^ and chicken^75^. A previous study reported that the acetyl CoA metabolic network related genes (including ACACA) was down-regulated in obese individuals with type 2 diabetes compared to those with normal glucose tolerance^76^. These evidences support our speculation that down-regulated genes in Lori-Bakhtiari breed might indicate a shift in fat metabolism toward altered fatty acid composition. However, analyzing fatty acid profile of tail fat depots in these breeds is warranted to validate this hypothesis.

One of the important significantly enriched pathways was “extracellular matrix (ECM)-receptor interaction”, in which up-regulation of ITGA11, TNC and some members of collagens family (including COL1A1, COL1A2, COL6A3 and COL6A6) were observed in Zel breed. All the genes were members of orange module (Fig. 6), suggesting that they are co-regulated to form molecular networks. ECM-receptor interaction has been reported as enriched pathway in DEGs between fat-tail tissue of Guangling Large Tailed and Small Tailed Han sheep^43^. A transcriptome study in adipose tissue of cattle highlighted the importance of ECM pathway in adipogenesis^77^. Down-regulation of genes encoding ECM has been shown during differentiation of human mesenchymal stromal-cells into adipocytes^78^. Thus, it is tempting to speculate that accumulation of less fat in tail of Zel breed is associated with up-regulation of collagen genes as well as other ECM-related genes.

Several DEGs (including COL1A1, COL5A1 and discoidin domain receptor tyrosine kinase 2 (DDR2)) involved in “Wnt signaling pathway” were down-regulated in Lori-Bakhtiari breed. The activation of “Wnt signaling pathway” can result in restriction of adipogenesis^79^. Besides, the orange module (Fig. 6) was also significantly enriched for “positive regulation of canonical Wnt signaling pathway”, suggesting that the expression of these genes might play an active role in regulating fat deposition in sheep tails.

In addition, down-regulated DEGs were predominantly over-represented in term of “branched-chain amino acid catabolic process”. The catabolism of branched chain amino acids profoundly impacts adipose fat cell accumulation so that it supplies over 40% of carbon skeleton needed for biosynthesis of fatty acids and other fatty acid-driven molecules. Nevertheless, the expression profile of genes controlling this process is recently shown to be dependent on the differentiation phase of adipocytes, repressing as the cell advances to full differentiation^80^. On the other hand, there are limited reports indicating that elevated circulating concentrations of these amino acids may serve as biomarkers of insulin resistance^81^. Therefore, down-regulation of DEGs related to the branched-chain amino acid catabolic process in Lori-Bakhtiari might have been resulted from taking samples at the end of the experiment, i.e. the full differentiation stage of adipose tissue or an adaptive mechanism to maintain insulin sensitivity in the fat-tail tissue. Alternatively, this process might have been differentially up-regulated in tail of the Zel breed as a result of increased storage of fatty acids in adipocytes as this process is stimulated with increased glucose availability.

Several DEGs related to “regulation of actin cytoskeleton” were down-regulated in Lori-Bakhtiari compared to Zel breed. Interestingly, decreased expression of the genes related to these processes occurred during adipocyte differentiation in sheep^82^. Also, the importance of this pathway in fat deposition and adipocyte regulation have been documented in longissimus dorsi muscle of bovine^83^ as well as in Ankole cattle breed^84^. These results suggest a link between down-regulation of these genes and deposition of greater amount of fat in Lori-Bakhtiari breed.

In addition, several TFs were found (based on AnimalTFDB database^85^) among the DEGs, of which seven were up-regulated (JUNB, distal-less homeobox 5 (DLX5), ATF3, ARID5, MYBL2, MAFF and ENSOARG00000011169), while four were down-regulated (estrogen receptors α (ESR1), SMAD family member 5 (SMAD5), neuronal PAS domain protein 2 (NPAS2) and myocyte enhancer factor 2A (MEF2A)) in Lori-Bakhtiari breed. Functional crucial roles of four of the seven up-regulated TFs that were presented in PPI network (Fig. 5), were discussed above. Of the four down-regulated TFs, two TFs (ESR1 and MEF2A) were observed in PPI network (Fig. 6). ESR1 is involved in energy balance^86^ and previous studies have reported a clear relationship between ESR1 expression level^87,88^ as well as it’s polymorphisms^89^ with obesity. MEF2A regulates Glucose transporter type 4 (GLUT4)^90^, which is the predominant glucose transporter mainly expressed in adipose tissue. Down-regulation of GLUT4 gene has been reported in adipose tissue, under obese conditions in both humans and rodents^91^. These were in accordance with down-regulation of DEGs related to branched chain amino acid catabolism in terms of the common effect that they have on insulin resistance. The present findings indicate that the transcriptome changes of these TFs may activate the corresponding down-stream genes ultimately regulating in fat deposition in tail of sheep. Moreover, four up-regulated and two down-regulated TFs presented in PPI network seem particularly promising for further studies on fat deposition.

## Conclusions

In this study, two Iranian sheep breeds with the fat- and thin-tail were compared through RNA-Seq data. Some specific candidate pathways containing genes involved in lipid deposition were identified. Results suggested that in addition to pathways affecting lipid metabolism, a series of enriched functional terms related to “response to interleukin”, “MAPK signaling pathways”, “Wnt signaling pathway”, “ECM-receptor interaction”, “regulation of actin cytoskeleton” and “response to cAMP” may contribute to the deposition of fat in the tail of Lori-Bakhtiari sheep, through interacting with pathways related to lipid metabolism. Moreover, PPI network analysis showed that there was a close interaction among the DEGs. Results suggested that four modules, which were significantly enriched with GO terms as well as KEGG pathways related to lipid metabolism, were also found in PPI networks. This means that members of these modules can be considered as important candidate genes in tail fat metabolism in sheep. Several genes were found to be differentially expressed in the sheep breeds, including ZFP36, JUNB, RARRES2, ADIRF, AFT3, ARID5A, MYBL2, GADD45B, SCD, FASN, ACACA, CPT2, ELOVL5, ELOVL6, ACSL1, EHHADH, HSD17B12, ACLY, COL1A1, COL1A2, ITGA11, TNC, COL6A3, ESR1 and MEF2A, which were directly or indirectly important in fatty acid metabolism. Differential expression of related genes may promote fat deposition in the tail of Lori-Bakhtiari, compared with the Zel breed. Overall, our findings suggested that the regulation of adipocyte differentiation might be different in these breeds. It is obvious that understanding the specific regulation pathways of fat deposition is difficult to verify solely by gene expression profiling. Therefore, further work based on the results from this study are required to uncover the exact genetic mechanisms of fat deposition in the tail of sheep.

## Supplementary information

Supplementary File S1

Supplementary File S2

Supplementary File S3

Supplementary File S4

Supplementary File S5

Supplementary File S6

Supplementary File S7
