# Supplementary material for: Deep transcriptome analysis using RNA-Seq suggests novel insights into molecular aspects of fat-tail metabolism in sheep
Source: Sci Rep. 2019 Jun 24;9:9203. doi: 10.1038/s41598-019-45665-3 (PMC6591244; doi:10.1038/s41598-019-45665-3)
Supplement: Supplementary file 3 — Supplementary File S3 [file 41598_2019_45665_MOESM3_ESM.docx]

**Deep transcriptome analysis using RNA-Seq suggests novel insights into molecular aspects of fat-tail metabolism in sheep**

Mohammad Reza Bakhtiarizadeh^a^*, Abdolreza Salehi^a^, Ali Assadi-Alamouti^a^, Rostam Abdollahi-Arpanahi^a^, [Seyed Alireza Salami](https://www.nature.com/articles/s41598-017-15816-5#auth-4)^b^

^a^ Department of Animal and Poultry Science, College of Aburaihan, University of Tehran, Tehran, Iran

^b^ University of Tehran, Tehran, Iran

* Corresponding author. E-mail address: mrbakhtiari@ut.ac.ir

**
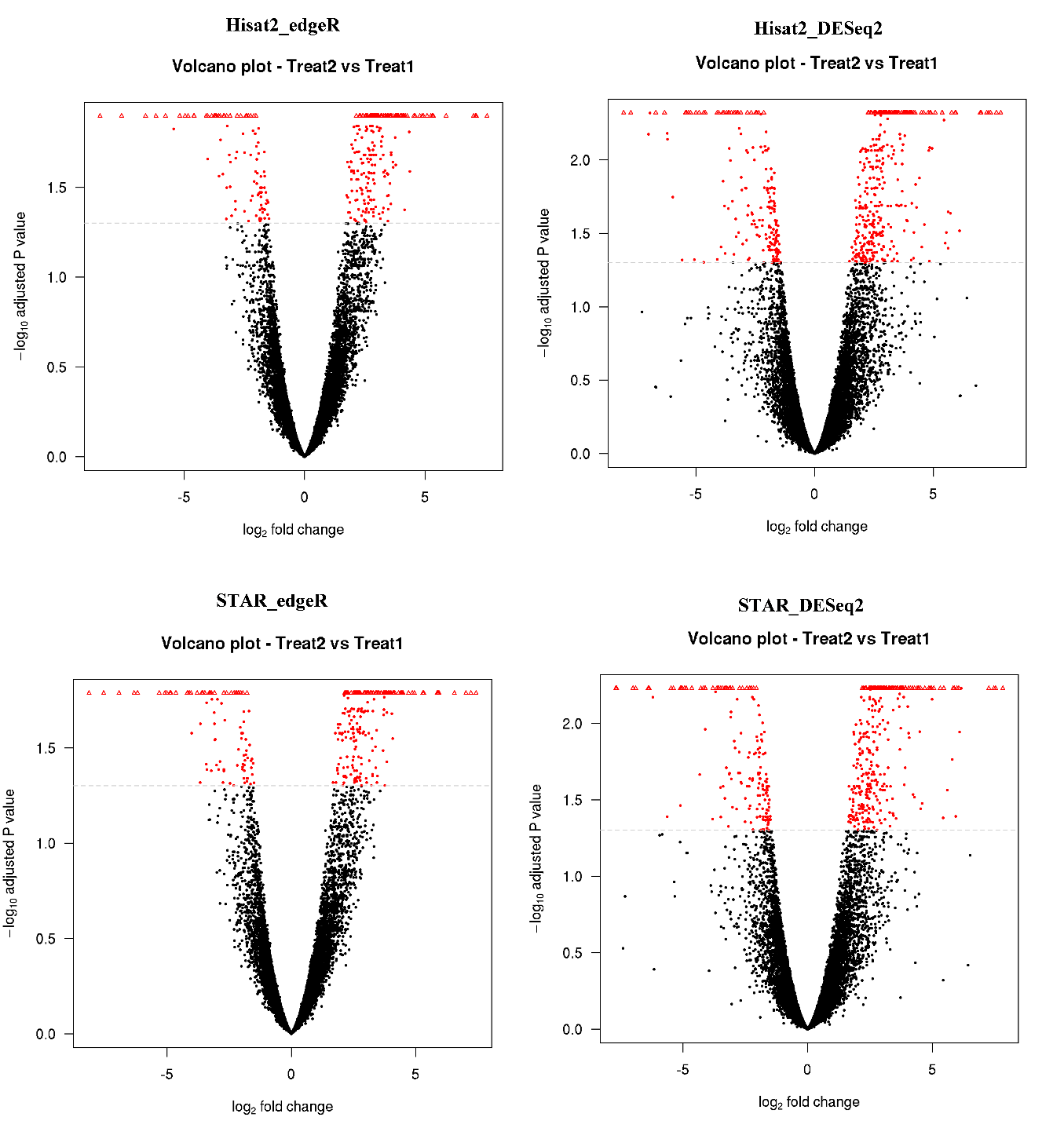
Supplementary File S3.** Volcano plot of differentially expressed genes between two sheep breeds for all four methods.
